# *Acinetobacter baumannii* does not affect *Candida albicans* Susceptibility to Fluconazole *in vitro*

**DOI:** 10.17912/micropub.biology.001817

**Published:** 2025-09-30

**Authors:** Autumn Perley, Allie Conner, Melody Neely, Robert T. Wheeler

**Affiliations:** 1 Molecular & Biomedical Sciences, University of Maine, Orono, Maine, United States; 2 Molecular & Biomedical Sciences, University of Maine; 3 Graduate School of Biomedical Sciences and Engineering, University of Maine, Orono, Maine, United States

## Abstract

*Candida albicans* is the most common human fungal pathogen and is difficult to treat due to the scarcity of and increasing resistance to fungicidal drugs. *C. albicans* is commonly isolated with bacterial pathogens such as *Pseudomonas aeruginosa* and *Acinetobacter baumannii* that also cause multidrug-resistant hospital-acquired infections. We have recently described how the presence of *P. aeruginosa* makes the antifungal drug fluconazole (FLC) more effective and switches it from fungistatic to fungicidal against *C. albicans* , a process sensitive to iron chelation. Although different *P. aeruginosa* isolates enhance fluconazole sensitivity, it is unknown if other bacterial species have a similar effect. *A. baumannii* is similar to *P. aeruginosa* : it is gram-negative, drug resistant, produces siderophores and causes intractable nosocomial infections. In this study, we investigated if *A. baumannii* has a similar bacterial-drug synergy against *C. albicans* . We found that *A. baumannii* shows little to no enhancement of FLC against *C. albicans in vitro.* Our work indicates that not all opportunistic bacterial pathogens have the same effect on *Candida* drug susceptibility as *P. aeruginosa* . It further suggests that *P. aeruginosa* produces unique molecules that drive its interactions with *C. albicans* and contribute to the clearance of *C. albicans* in the presence of FLC. This work addresses the need to understand how microbe-microbe interactions regulate virulence and antimicrobial susceptibility of fungal pathogens, a still poorly understood aspect of infection and colonization.

**Figure 1. A. baumannii does not synergize with fluconazole against C. albicans:**
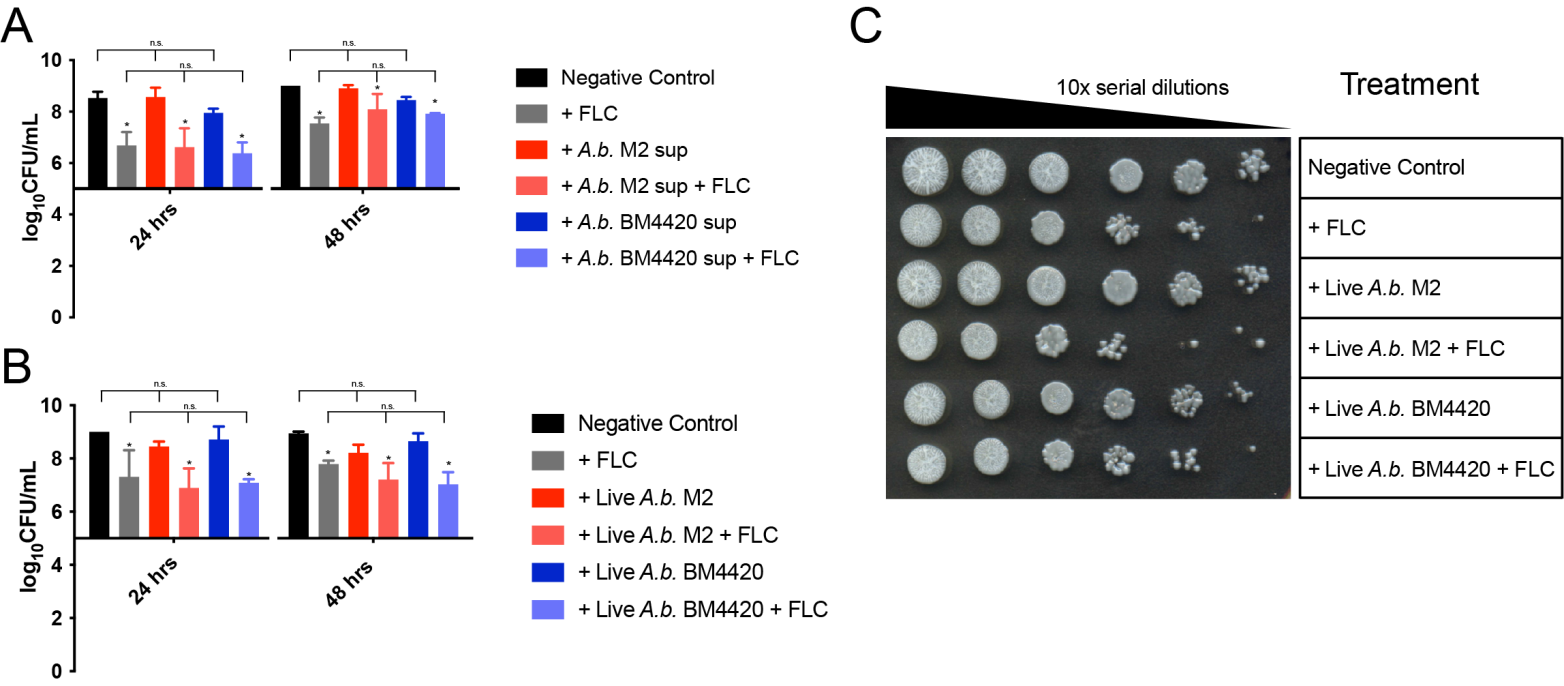
(A) Fluconazole is not synergistic with *A. baumannii* M2 or BM4420 supernatant against *C. albicans* *in vitro* . (B) Fluconazole is not synergistic with live *A. baumannii* M2 or BM4420 against *C. albicans* *in vitro* . (C) *C.***** *albicans* + *A. baumannii* + FLC shows no fungicidal effect after coculture. *C. albicans***** and**** *A. baumannii* (or *A. baumannii* supernatant) were inoculated at 2 x 10 ^6^ /mL and 2 x 10 ^5^ /mL starting concentrations, respectively, and FLC was added at 12.5 µg/mL. Drops (3 µL) of serial 10x dilutions of cocultures grown for 24 or 48 hours were plated on YPD containing antibiotics and LB containing FLC. Each graph is representative of 3 biologically- independent experiments and the image is representative of duplicates run for 3 biological replicates. Statistical tests are described in Materials & Methods. Asterisks indicate a significant (p<0.05) difference in fungal viability upon FLC treatment. N.s. indicates comparisons in which there was no significant difference (p>0.05).

## Description

*Candida albicans* is a fungus that colonizes most people as part of their natural microbiome (Berkow & Lockhart, 2017). *C. albicans* also causes both superficial and systemic infections, accounting for approximately 75-88% of all human fungal infections in the United States. *C. albicans* associates with and causes infections in several body sites, including the respiratory, gastrointestinal, and genitourinary tracts, as well as burn wounds. It is the 4th most common nosocomial (hospital-acquired) pathogen in the US, with systemic infections of particular concern for those who are immunocompromised (Berkow & Lockhart, 2017; Mayer, Wilson, & Hube, 2013).

The antifungal drug fluconazole (FLC) is one of the most frequently prescribed for *Candida* infections. It blocks the production of ergosterol, a critical component of the fungal cell membrane, which results in arrested cell growth (Cha & Sobel, 2004; Govindarajan, Bistas, Ingold, Patel, & Aboeed, 2025; Zervos & Meunier, 1993). FLC is typically fungistatic, but in the presence of *P. aeruginosa* , it has fungicidal activity both *in vitro* and *in vivo* (Hattab, Dagher, & Wheeler, 2022). This has been tied to iron piracy by *Pseudomonas* , and is a robust phenomenon seen among multiple *P. aeruginosa* and *C. albicans* isolates (Hattab et al., 2022). Iron is an important micronutrient that is required by bacteria and fungi for many cellular processes including the generation of ATP in the electron transport chain, DNA repair and replication, and other physiological activities (Cook-Libin, Sykes, Kornelsen, & Kumar, 2022; Law & Tan, 2022).

*Acinetobacter baumannii* , like *P. aeruginosa* , is a Gram-negative opportunistic pathogen (Cook-Libin et al., 2022). They both belong to the ESKAPE pathogen class (along with *Klebsiella* , *Escherichia* , *Staphylococcus* and *Enterococcus* ) which combines the leading causes of nosocomial infections in the world (Santajit & Indrawattana, 2016). *A. baumannii* infects sites commonly colonized with and infected by *C. albicans* , including the respiratory tract, urinary tract, burn wounds, and the bloodstream (Cook-Libin et al., 2022; Kostoulias et al., 2016). Notably, *P. aeruginosa* and *A. baumannii* both produce iron-chelating siderophores that play a role in *P. aeruginosa* - *C. albicans* interactions during FLC treatment, suggesting they might have similar cross-kingdom interactions (Hattab et al., 2022; Ghavanloughajar, Elmassry, Brown, & Hamood, 2025).

To determine if *C. albicans* has similar antagonistic interactions with other co-occurring ESKAPE bacteria with respect to antifungal susceptibility, we sought to determine if *A. baumannii* affects FLC activity against *C. albicans* . Since *A. baumannii* secretes several factors into the media (Dorsey et al., 2004; Jin et al., 2011), we first tested if the supernatant from *A. baumannii* overnight cultures enhances FLC activity against *C. albicans* , as does the conditioned media of *P. aeruginosa* (Hattab et al., 2022). We collected supernatant from cultures of M2 and BM4420, two different clinical strains of *A. baumannii* , grown overnight in LB broth media (Niu, Clemmer, Bonomo, & Rather, 2008; Ploy, Denis, Courvalin, & Lambert, 2000). Given the significant genetic heterogeneity of *A. baumannii* isolates, the use of two isolates served to identify any strain-specific interactions: the M2 isolate is a genetically-tractable clinical isolate that offers the potential for subsequent genetic manipulation, while BM4420 is a multidrug resistant isolate from a different continent (Harding et al., 2013; Niu et al., 2008; Ploy et al., 2000; Zimbler et al., 2009). The supernatants were filter-sterilized and added to *C. albicans* in YPD plus FLC at a 1:1 dilution, followed by growth for two days at 30°C. Cultures were serially diluted at 24- and 48-hours post-inoculation and spotted to quantify the concentration of live colony-forming units per milliliter (CFU/ml). Each culture was also spotted onto control plates (YPD + FLC) to ensure that there was no contamination with live bacteria in any cultures. Supernatants from neither isolate affected growth and viability of *C. albicans* , either alone or in the presence of FLC ( Fig. 1A ). This suggests that secreted factors from *A. baumannii* do not affect FLC tolerance.

Since supernatants from *P. aeruginosa* retain only a portion of the potency of live *P. aeruginosa* to enhance susceptibility to FLC, we sought to test if co-culture with live *A. baumannii* affects *C. albicans* susceptibility. First, we confirmed that our *A. baumannii* isolates grow well in YPD media, which is optimal for *C. albicans* growth. Then, we set up co-cultures with fungal and bacterial cells in the presence or absence of FLC, allowing them to grow for two days and measuring fungal viability (as CFU/ml) at 24- and 48-hours post-inoculation. Each culture was also spotted onto control plates (YPD + FLC) to ensure that there was no contamination with live bacteria in any fungi-only cultures. Co-culture with *A. baumannii* did not affect fungal susceptibility to FLC after either 24 or 48 hours of culture, and this was the case for both clinical isolates of *A. baumannii* ( Fig. 1B ). A spot plate from a representative experiment illustrates the feasibility of using serial dilutions for co-culture CFU quantification ( Fig. 1C ).

This project sought to determine if other bacterial opportunistic pathogens can affect drug susceptibility of *C. albicans* . The lack of any effect of *A. baumannii* on FLC susceptibility suggests that the mechanisms determining enhanced *C. albicans* susceptibility induced by *P. aeruginosa* are not ubiquitous among gram-negative, iron-chelating bacterial pathogens. This contrasts with an apparently broad activity of *P. aeruginosa* against *Candida* spp. fungi, which also increases the FLC susceptibility of *Candida glabrata* , a species quite distant evolutionarily from *C. albicans* (Hattab et al., 2022). Further, since both *P. aeruginosa* and *A. baumannii* produce potent siderophores, our results also suggest that siderophore-mediated iron chelation is not sufficient for enhancing FLC susceptibility. One caveat to this work is that we only tested one type of media, and sometimes these interactions are media-dependent (Ghavanloughajar, Elmassry, Brown, & Hamood, 2025).

Our long-term goals include determining what molecular mechanisms are responsible for the synergy of FLC with bacteria like *P. aeruginosa* and understanding how such bacterial-fungal interactions regulate drug susceptibility during human infection and treatment.

## Methods

**Bacterial strains and culturing**

The strains used were *C. albicans* SC5314, streaked from frozen stocks to YPD (1% Bacto Yeast extract (Gibco USA), 2% Bacto Peptone (Gibco USA), 2% Bacto Agar (Gibco USA), 2% dextrose (Thermofisher); autoclaved 30 min at 121C). *A. baumannii* strains M2 and BM4420 are de-identified patient isolates(Niu et al., 2008; Ploy et al., 2000); they were streaked from frozen glycerol stocks to LB (10 g/liter Bacto tryptone (Gibco USA), 5 g/liter Bacto yeast extract (Gibco USA), 2% Bacto Agar (Gibco USA), 10 g/liter sodium chloride (Thermofisher). *C. albicans* was pre-cultured in 3 mL of YPD liquid for 24 hours overnight on a roller drum (New Brunswick Scientific TC-7) at 30°C for 24 hours. Cell concentration after culturing was measured by OD _600_ and was 1-4*10 ^8^ cells/ml. Note that OD _600_ =1 is equivalent to 10 ^7^ cells/ml. *A. baumannii* was pre-cultured in LB at 30°C for 24 hours. Cell concentration after culturing was measured by OD _600_ and was 1-4*10 ^9^ cells/ml. Note that OD _600_ =1 is equivalent to 10 ^8^ cells/ml. *A. baumannii* and *C. albicans* cultures were diluted 1:1000 from each overnight culture into fresh YPD liquid media with the organisms at final concentrations of 2 x 10 ^6^ /mL and 2 x 10 ^5^ /mL, respectively. Spent supernatant from *A. baumannii* pre-culture in LB was prepared by a combination of high-speed centrifugation followed by filter sterilization, as follows. Overnight *A. baumannii* culture was pipetted into 50 mL conical tubes and centrifuged at 13,855 x g for 15 minutes (using the BioSEAL F13-14x50cy rotor in the Thermo Scientific Sorvall X Pro Series Centrifuge). The supernatant was then triple-filtered using 0.2 µm syringe filters (Nalgene Prefilter Plus GFP + 0.2 µm CA; Thermo Scientific) and stored protected from light at 6°C. In cultures with spent supernatant, the supernatant was boiled for ten minutes, then diluted 1:1 with fresh YPD. Fluconazole was added at 12.5 µg/mL, from a stock of 2 mg/mL in water. This is significantly above the MIC _50_ for strains and limits any experimental variability due to slight differences in drug concentration. Co-cultures were then grown on a roller drum at 30°C for 48 hours.

**Quantification of fungal viability**

After growth for 24 or 48 hours, aliquots were obtained from each culturing condition, diluted in 10-fold serial dilutions in sterile phosphate-buffered saline (PBS) (137 mM NaCl, 2.7 mM KCl, 10 mM Na _2_ HPO _4_ O, and 2 mM KH _2_ PO _4_ O), and spot-inoculated (3 μl/spot) in duplicate onto YPD agar + antibiotics (10 μg/mL gentamicin (MP Biomedicals), 25 IU/mL penicillin (VWR USA), and 25 μg/mL streptomycin (VWR USA)) and LB + FLC agar plates. The 24 and 48-hour plates grew at 37°C overnight, then were scanned and counted for viable CFU.

**Statistical Analyses and Figure Preparation**

Data shown is representative of at least 3 individual replicates. All plots were graphed and statistical comparisons (one-way ANOVA with multiple comparisons testing) were calculated using GraphPad Prism Version 6.07 (GraphPad Software, Inc., La Jolla, CA). Figures were created in Illustrator (Adobe, Inc.).
